# Prosthetic Knee Joint Infection Secondary to *Granulicatella adiacens*: A Case Report

**DOI:** 10.1155/crdi/5407160

**Published:** 2025-02-26

**Authors:** Edward Lovering, Farhang Alaee, Michelle Bahrain

**Affiliations:** ^1^Department of Internal Medicine, Medstar Health Internal Medicine Residency Program, Baltimore, Maryland, USA; ^2^Department of Orthopedics, Medstar Franklin Square Medical Center, Baltimore, Maryland, USA; ^3^Department of Infectious Disease, MedStar Franklin Square Medical Center, Baltimore, Maryland, USA

**Keywords:** *Granulicatella adiacens*, nutritionally variant streptococci, prosthetic joint infection

## Abstract

*Granulicatella adiacens* is an uncommon cause of prosthetic joint infection (PJI). Here, we report a case of *Granulicatella adiacens* prosthetic knee joint infection in a patient who was previously managed for *Granulicatella* bacteremia 10 months earlier. The infection was managed with a planned two-stage surgical revision and prolonged IV antibiotics. This case underscores the rare pathogenic potential of a typically benign commensal organism, highlighting the importance of considering atypical pathogens in common clinical presentations.

## 1. Introduction

*Granulicatella* species, formerly known as nutritionally variant streptococci, are gram positive cocci which make up part of the normal oral flora in humans [1]. *Granulicatella* is a rarely isolated pathogen in humans and is not commonly associated with prosthetic joint infections (PJIs) in the literature. In the following, we report the case of a 66-year-old female with a prosthetic knee joint infection secondary to *Granulicatella adiacens.*

## 2. Case Presentation

A 66-year-old female presented to routine outpatient orthopedic clinic due to worsening pain and discomfort in the left knee for several months. She had associated swelling over the joint and difficulty mobilizing the knee. On physical exam, she was found to have global swelling of her left knee joint, which was warm and erythematous, with pain present on passive movement.

The patient had a past surgical history of aortic bioprosthetic valve replacement 5 years prior, alongside left-sided total knee arthroplasty (TKA) 15 years prior and right TKA 8 years prior.

Notably, the patient was hospitalized 10 months earlier at an outside hospital for fever and shortness of breath. Investigation demonstrated high grade and sustained *Granulicatella adiacens* bacteremia in four sets of blood cultures. She underwent a transesophageal echocardiogram, which was negative for vegetations. PET/CT imaging was not available. She was treated for *Granulicatella adiacens* bacteremia with a 6-week course of IV vancomycin and gentamicin.

She underwent left knee aspiration in the outpatient clinic, which yielded 30 mL of cloudy fluid, and the patient was admitted to the hospital for management of possible septic arthritis.

Upon admission to hospital, the patient's vital signs were normal with absence of fever. Initial laboratory work demonstrated elevated C-reactive protein of 57 (reference range: 0.00–10.00 mg/L) and elevated erythrocyte sedimentation rate of 96 (reference range: 0–30 mm/hr). Initial blood cultures demonstrated no growth.

Synovial fluid analysis demonstrated 111,326 total nucleated cells with 96.1% neutrophils. Synovial fluid was positive for alpha defensin by ELISA on synovasure lateral flow test. Bacteria was cultured using BACTEC blood culture media and upon growth of the isolate, the VITEK 2 system was used for isolate identification. Antimicrobial sensitivity testing was performed and interpretated per Clinical and Laboratory Standards Institute (CLSI) guidelines (Table 1).

The patient underwent stage I revision knee arthroplasty with removal of all knee replacement components and removal of the distal femoral locking plate, with successful placement of articulating spacer. Tissue cultures from the operating room were negative as were admission synovial fluid cultures. Two sets of peripheral blood cultures demonstrated no growth at day 5. Transthoracic echocardiogram demonstrated no vegetations. The patient recovered well from surgery and was discharged home on IV vancomycin with the plan for stage II revision once antibiotic course completed.

## 3. Discussion

PJI after TKA is an uncommon but serious complication, occurring at an annual incidence of approximately 2%. The most frequently identified pathogen in these infections is *Staphylococcus aureus.* [2] *Granulicatella adiacens*-related PJIs are even rarer, with only eight reported cases in the literature: four involving prosthetic knee joints [3–6] and four involving prosthetic hip joints [5, 7, 8]. In addition, there have been three reported cases of septic native arthritis of the knee caused by *Granulicatella adiacens* following invasive procedures such as arthrocentesis [9–11].

Granulicatella species present a diagnostic challenge, as they are fastidious bacteria with specific nutritional requirements. Successful growth and identification typically require the presence of growth factors such as pyridoxal and L-cysteine in the culture media [1]. Synovasure lateral flow test was used in the case above to help establish the initial diagnosis of PJI. This test can identify alpha defensin within the synovial fluid and has been well validated as part of the diagnostic criteria for PJI [12–14]. The initial growth of the isolate was made through culturing of synovial fluid in BACTEC blood culture media. This technique has previously been shown to demonstrate higher diagnostic yield in septic arthritis compared with standard synovial culture on agar plates alone [15]. The eventual identification of the organism was made through the automated VITEK 2 system.

Other previous cases of *Granulicatella adiacens* PJI (hip and knee) have been diagnosed through molecular and biochemical methods; with five cases identified through 16S rRNA gene sequencing of synovial fluid [3–6, 8]; two cases identified through matrix-assisted laser desorption/ionization time-of-flight mass spectrometry systems (MALDI-TOF MS) of bacterial colonies from synovial fluid grown in blood culture media [5, 7]; and one case which was identified through both 16S rRNA gene sequencing and MALDI-TOF MS [5].

For a considerable time, 16S rRNA sequencing has been regarded as the benchmark for detecting this isolate [16] and was pivotal in initially distinguishing this species from nutritionally deficient streptococci [1]. However, 16S rRNA gene sequencing is associated with longer turnaround times of two to three days and may only be accessible at certain institutions. A study that compared various MALDI-TOF systems to a reference standard of 16S rRNA gene sequencing for identifying Granulicatella species showed that the MALDI-TOF Vitek MS system is a dependable and reliable method for rapidly diagnosing Granulicatella species [17].

*Granulicatella adiacens* is a common commensal of the oral cavity, and three cases noted prior dental procedures, highlighting the potential hematogenous spread from the oral cavity to the joint space [4, 5, 7]. In the case reported above, the patient had no dental history but had a prior history of bacteremia secondary to *G. adiacens* 10 months prior to presentation, which was appropriately treated with IV vancomycin and gentamicin. She reported that her knee pain began toward the end of therapy with antibiotics, but she did not report pain to her providers then. In this case, she likely had hematogenous seeding of the knee previously, which recurred off antibiotics. Most previous cases were treated with two stage prosthesis exchange coupled with prolonged course of antibiotics; only one case was successfully managed without removal of prosthesis [5].

Antibiotic susceptibilities for *G. adiacens* can be challenging to obtain given the difficulties in growing the isolate. There is currently no global consensus on the antibiotic regiment for *G. adiacens* PJI given the limited number of cases available. However, previous research into antibiotic sensitivities of isolated *G. adiacens* species has shown approximately 40% susceptibility to penicillin, approximately 50% susceptibility to ceftriaxone and 100% susceptibility to vancomycin [18]. Ultimately, as with any infection, achieving appropriate source control is paramount in controlling the infection.

## 4. Conclusion

In summary, PJIs caused by *G. adiacens* remain a rare, and likely, under reported infection. It poses a major diagnostic challenge; however, techniques such as 16S rRNA gene sequencing and MALDI-TOF prove to be valuable resources in confirmation of this organism and will likely aid clinicians in the future. Given the scarcity of this condition and the lack of established treatment guidelines, case reports like this one can offer valuable insights that can help guide future healthcare providers in managing similar cases.

## Figures and Tables

**Table 1:** Culture and sensitivity data for *Granulicatella adiacens* species isolated from knee synovial fluid.

Drug	MIC dilution	MIC interpretation
Cefepime	4	Resistant
Ceftriaxone	2	Intermediate
Erythromycin	> 2	Resistant
Levofloxacin	< 1	Sensitive
Meropenem	< 0.25	Sensitive
Penicillin	1	Intermediate
Vancomycin	1	Sensitive
Clindamycin	0.5	Intermediate

*Note:* Interpretation of MIC and antimicrobial sensitivity was based off Clinical and Laboratory Standards Institute (CLSI) guidelines.

Abbreviation: MIC, minimum inhibitory concentration.

## Data Availability

Data sharing is not applicable to this article as no new data were created or analyzed in this study.
